# Prepubertal onset of slipped capital femoral epiphysis associated with hypothyroidism: a case report and literature review

**DOI:** 10.1186/s12902-017-0210-6

**Published:** 2017-09-18

**Authors:** Saori Kadowaki, Tomohiro Hori, Hideki Matsumoto, Kaori Kanda, Michio Ozeki, Yu Shirakami, Norio Kawamoto, Hidenori Ohnishi, Toshiyuki Fukao

**Affiliations:** 1 Department of Pediatrics, Graduate School of Medicine, Gifu University, 1-1 Yanagido, Gifu, Gifu 501-1194 Japan; 2 Department of Pediatrics, Kibogaoka Medical and Support Center for Children, 1816-1 Noritake, Gifu, Gifu 502-0931 Japan

**Keywords:** Atrophic thyroiditis, Endocrinology, Hypothyroidism, Pediatrics, Slipped capital femoral epiphysis

## Abstract

**Background:**

Slipped capital femoral epiphysis (SCFE) is a common hip disorder characterized by displacement of the capital femoral epiphysis from the metaphysic through the femoral epiphyseal plate. SCFE usually occurs during puberty, with obesity a common risk factor. We experienced a rare case of SCFE associated with hypothyroidism in a prepubescent patient who was not obese.

**Case presentation:**

The patient was an 8-year-old boy suffering from bilateral SCFE with hypothyroidism. The patient’s growth had started to slow at 4 years of age, and at 8 years he was of short stature. During his evaluation for SCFE management, primary hypothyroidism was diagnosed due to the presence of anti-thyroid peroxidase and anti-thyroglobulin antibodies. After the patient was treated for hypothyroidism, which improved his thyroid function, surgery was performed for bilateral SCFE.

**Conclusions:**

Among the 42 patients with SCFE associated with hypothyroidism in the literature, most SCFE occurred during puberty or in adults with delayed epiphyseal closure. Only two patients (4.8%), including the present patient, were ≤9 years old. Although being overweight or obese is common for patients with SCFE associated with hypothyroidism (76.0%), it was not observed in the present case. Persistent hypothyroidism, however, may be a risk factor for SCFE even before puberty and without obesity.

## Background

Slipped capital femoral epiphysis (SCFE) is defined as posterior and inferior slippage of the proximal femoral epiphysis on the metaphysis (femoral neck), which occurs through the epiphyseal plate (growth plate) [1, 2]. SCFE, a rare disease, is known to be strongly associated with obesity. Most patients develop SCFE during puberty. The majority of SCFE cases are idiopathic, although, atypically, SCFEs may be due to an endocrine disorder, renal failure, osteodystrophy, or radiation therapy. The current incidence of SCFE in children 8–15 years of age ranges from 0.33/100,000 to 24.58/100,000, depending on sex and ethnicity [3]. From 1976 to 2002 in Japan, the incidence of SCFE in those 10–14 years of age increased from a range of 0.3–0.5 to 2.22/100,000 in boys and from 0.05–0.08 to 0.76 in girls [4]. Thus, the incidence has increased approximately 5-fold in boys and 10-fold in girls, bringing it closer to the figures in other countries. The main cause for the increased numbers of SCFE in Japanese children is thought to be obesity.

Rarely, SCFE is an important complication of hypothyroidism that often develops during childhood. We describe a case of hypothyroidism accompanied by SCFE in a prepubertal patient. We also review previously reported cases of SCFE associated with hypothyroidism.

## Case presentation

**Fig. 1:**
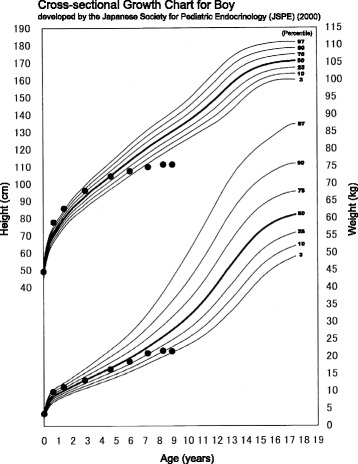
Growth chart for the patient. Serial height and weight measurements of the patient were plotted using the standard growth chart (developed by the Japanese Society for Pediatric Endocrinology)

**Fig. 2:**
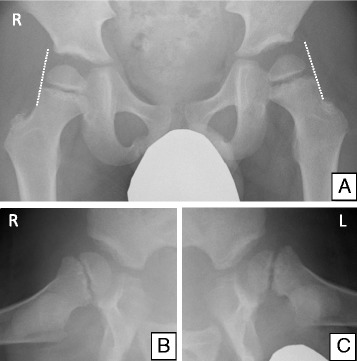
Radiological findings of the patient. Radiographs of the hip joint show Trethowan’s sign bilaterally in the frontal view (**a**) and a posterior tilting angle of 25° on the right side (**b**) and 34° on the left side (**c**) in the Lauenstein view

An 8-year-old boy presented with pain in his left hip joint that had persisted for several months. His height was 112.5 cm (−2.93 standard deviation), and his weight was 21.5 kg, from which body mass index was calculated to 67.1 percentile for age and sex. His growth had started to slow at 4 years of age (Fig. 1). He presented with hirsutism, dry skin, and bradycardia. Hip joint radiography revealed Trethowan’s sign bilaterally in the frontal view and a posterior tilting angle of 34° on the left side and 25° on the right side in the Lauenstein view (Fig. 2). Magnetic resonance imaging (MRI) of the hip joint showed separation of the proximal metaphysis of the femur. Based on these findings, the patient was diagnosed with chronic bilateral SCFE (mild on the right side, moderate on the left side).

Blood tests revealed hepatic dysfunction and hypercholesterolemia, with a very low free thyroxine level of 0.10 ng/dl and a very high thyroid-stimulating hormone (TSH) level of 1789 μIU/ml. The anti-thyroid peroxidase antibody level was >600 IU/ml, and the anti-thyroglobulin antibody level was 1720 IU/ml. At the age of 8 years 7 months, the bone age (assessed by the Tanner–Whitehouse 2 radius, ulna, short bones method) was 3 years 4 months. Ultrasonography showed marked atrophy of the thyroid, and color-flow Doppler sonography revealed decreased thyroid blood flow. Cardiac ultrasonography disclosed slight retention of pericardial fluid. MRI scans of the head showed that the anterior lobe of the pituitary gland was enlarged to a height of 17 mm. Although the pituitary gland displaced the optic chiasm superiorly, there were no visual acuity or field abnormalities. Hyperplasia of the anterior lobe of the pituitary gland caused by the overproduction of TSH was suspected. Therefore, atrophic thyroiditis was diagnosed.

For SCFE treatment, the patient was admitted and kept at rest, with the lower limbs in traction. Oral levothyroxine therapy was initiated with a low dose, followed by gradual dose increases. About 2 months later, the free thyroxine levels had increased to the normal range. In addition, the hirsutism, dry skin, and bradycardia had diminished, and his growth velocity had improved. MRI scans of the head showed reduction of the swollen pituitary gland. An arginine stimulation test (0.5 g/kg infused intravenously over 30 min) showed a normal growth hormone response (peak 7.26 ng/ml). Also, a human corticotropin-releasing hormone stimulation test (1.5 μg/kg infused intravenously) showed a normal adrenocorticotropic hormone response (peak 48.4 pg/ml) and cortisol response (peak 14.4 μg/dl). After 2 months of thyroxine treatment, the patient’s thyroid levels had stabilized, at which time surgery was performed for bilateral SCFE.

The CARE guidelines were followed in this case.

## Discussion and conclusions

We describe herein an 8-year-old patient with bilateral SCFE. He was diagnosed with hypothyroidism during the examination for possible SCFE after 4 years of slow growth. We also reviewed the literature for other cases of SCFE with hypothyroidism versus those with SCFE alone.

An epidemiological study of 314 patients with SCFE in Japan showed that most cases occurred during adolescence. Only seven patients had developed SCFE after the age of 20 years, and around one in every eight SCFE cases occurred before the age of 9 years. Endocrine abnormality was present in only 3 of 307 patients <20 years of age, whereas all 7 patients aged ≥20 years had an endocrine disease [4]. The proximal epiphysis of the femur generally closes between 15 and 17 years of age, after which SCFE does not occur. If epiphyseal closure is delayed because of the influence of an endocrine disease (e.g., hypothyroidism, hypogonadism), however, SCFE may occur even during adulthood. Hence, it is widely recognized that endocrine disease should be taken into consideration in cases of adulthood SCFE.

In addition to our current case, we reviewed the clinical features of 42 cases of SCFE with hypothyroidism from 24 reports published after 1980 (Table 1) [5–28]. The age of onset ranged from 7 to 28 years (mean 13.5 years, median 13 years). The onset of SCFE with hypothyroidism was commonly during adolescence. Accordingly, five patients (11.9%) had disease onset at ≥18 years. Only two patients (4.8%), including our own, had disease onset at ≤9 years of age. Thus, our case is a rare example of SCFE with hypothyroidism occurring prior to puberty.

**Table 1:** Reported cases of SCFE associated with hypothyroidism

Publication year [reference]	Age/Sex	Height^b^	Weight^a^	Diseased side
2004 [5]	7/M	short stature	obese or overweight	bilateral
Present case ^c^	8/M	short stature	normal body weight	bilateral
1984 [6]	10/F	N/A	N/A	right
2010 [7]	10/F	N/A	obese	bilateral
1984 [6]	11/M	short stature	N/A	bilateral
2004 [5]	11/F	short stature	obese or overweight	unilateral
2004 [5]	11/F	short stature	obese or overweight	unilateral
1993 [8]	11/F	normal stature	overweight	left
2004 [5]	12/M	short stature	obese or overweight	bilateral
2004 [5]	12/M	short stature	obese or overweight	unilateral
1993 [8]	12/M	normal stature	obese	left
2008 [9]	12/M	N/A	obese	bilateral
2016 [10]	12/M	normal stature	overweight	right
2004 [5]	12/F	short stature	obese or overweight	bilateral
1993 [8]	12/F	normal stature	obese	right
1980 [11]	12/F	N/A	N/A	bilateral
1980 [12]	12/F	short stature	N/A	left
1985 [13] ^c^	12/F	short stature	obese	left
2002 [14]	12/F	N/A	N/A	right
2014 [15]	12/F	short stature	overweight	left
1984 [6]	13/M	N/A	N/A	bilateral
1984 [6]	13/M	N/A	N/A	left
1984 [6]	13/M	short stature	N/A	bilateral
1992 [16] ^c^	13/M	N/A	N/A	left
2013 [17]	13/M	short stature	N/A	bilateral
2016 [18]	13/M	short stature	N/A	bilateral
1984 [6]	13/F	short stature	N/A	left
1984 [6]	13/F	N/A	N/A	right
2001 [19] ^c^	13/F	N/A	N/A	right
2010 [20]	13/F	short stature	normal body weight	left
2013 [21]	14/M	short stature	obese	right
1980 [12]	14/F	short stature	N/A	left
1994 [22]	14/F	short stature	normal body weight	right
2016 [10]	15/M	short stature	obese	left
2007 [23]	15/M	short stature	N/A	right
1993 [8]	15/F	short stature	overweight	right
2016 [18]	17/M	N/A	obese	bilateral
1988 [24] ^c^	18/F	short stature	overweight	left
2010 [25]	19/M	normal stature	normal body weight	left
1982 [26]	21/M	normal stature	normal body weight	right
2008 [27]	24/F	short stature	normal body weight	bilateral
2014 [28]	28/F	short stature	N/A	right

*M* male, *F* female, *N/A* not available

^a^According to growth charts developed by the National Center for Health Statistics in collaboration with the National Center for Chronic Disease Prevention and Health Promotion (http://www.cdc.gov/growthcharts, published in 2000, accessed 1 Sep. 2016), the 85th–95th body mass index (BMI) percentiles for age and sex were defined as overweight and ≥95th percentile as obese. Among patients aged 18 years or older, a BMI of 25 or more was defined as overweight, and a BMI of 30 or more as obese

^b^Short stature was defined as ≤3rd percentile

^c^Growth charts for Japanese individuals developed by the Japanese Society for Pediatric Endocrinology (http://jspe.umin.jp/medical/taikaku.html, published in 2000, accessed 1 Sep. 2016) was used for analysis in Japanese patients

We assessed the relation between the patient’s body weight and SCFE. It has been reported that SCFE patients with an age-specific body mass index in the ≥95th percentile accounted for 76.3% of cases, and those in the ≥85th percentile accounted for 92.5% of cases [29]. These data indicate that obesity is a risk factor for SCFE. As shown in Table 1, among the 42 patients with SCFE and hypothyroidism, body weight data were available for only 25, of whom 19 were obese or overweight (76.0%) (defined according to growth charts of the National Center for Health Statistics and National Center for Chronic Disease Prevention and Health Promotion) (Table 1). It is possible that myxedema contributed to their increased weight. These facts suggest that obesity is also a risk factor for SCFE with hypothyroidism. Our patient was not overweight, however, and so represents a rare example of SCFE with hypothyroidism from the viewpoint of body weight. It could also be presumed that the reports for the remaining 17 of the 42 patients did not refer to weight because the body weight of these individuals was proportional to their height. If this supposition is correct, non-obese patients would account for a relatively large proportion of those with SCFE associated with hypothyroidism.

Among the 42 patients with SCFE with hypothyroidism (Table 1), height was reported for 31. Short stature was seen in 25 patients (80.6%) (defined according to the growth charts referenced in Table 1), and 6 were of normal height. Because hypothyroidism during childhood inhibits growth, short stature indicates that hypothyroidism persists for several years before the induction of SCFE. Our patient presented with a 4-year period of reduced growth (Fig. 1). Persistent hypothyroidism may contribute to the development of SCFE.

The etiology of SCFE is poorly understood. In general, it is thought to develop in the presence of increased shearing force on the proximal epiphysis of the femur caused by obesity when growth hormones dominate over sex hormones during puberty [30]. Alternatively, thyroid hormone deficiency in children causes a delay in endochondral and intramembranous ossification and hypoplasia of the epiphyseal plate (growth plate). Thyroid hormone deficiency also induces inactivation of the growth hormone/insulin-like growth factor axis [31]. The effect of hypothyroidism on the growth plate has been observed in recent animal experiments in which swine suffering from hypothyroidism exhibited significantly decreased gene expression of proteoglycans and type X collagen. Such changes likely weaken overall epiphyseal strength and resilience, which could provide an insight into human orthopedic growth plate pathologies [32]. Moyer et al. [18] recommended that thyroid function screening be conducted in patients suffering from SCFE with an atypical presentation, which could include those presenting at <10 or >16 years of age, those with bilateral SCFE, and/or those whose height is ≤10% of normal for age and sex. In our patient, SCFE was triggered by prepubertal hypothyroidism, suggesting that hypothyroidism should be recognized as an independent risk factor for the development of SCFE.

In conclusion, thyroid function should be closely evaluated in patients with SCFE, especially those who are prepubescent, not obese, have a short stature for age and sex, and/or exhibit reduced growth.

## Abbreviations

MRI: Magnetic resonance imaging
SCFE: Slipped capital femoral epiphysis
TSH: Thyroid-stimulating hormone
